# The genome sequence of the large tortoiseshell, *Nymphalis polychloros* (Linnaeus, 1758)

**DOI:** 10.12688/wellcomeopenres.17196.1

**Published:** 2021-09-16

**Authors:** Konrad Lohse, Dominik Laetsch, Roger Vila, Darwin Tree of Life Barcoding collective, Wellcome Sanger Institute Tree of Life programme, Wellcome Sanger Institute Scientific Operations: DNA Pipelines collective, Tree of Life Core Informatics collective, Darwin Tree of Life Consortium

**Affiliations:** 1 Institute for Evolutionary Biology, University of Edinburgh, Edinburgh, UK; 2 Institut de Biologia Evolutiva (CSIC - Universitat Pompeu Fabra), Barcelona, Spain

**Keywords:** Nymphalis polychloros, large tortoiseshell, genome sequence, chromosomal

## Abstract

We present a genome assembly from an individual female *Nymphalis polychloros *(the large tortoiseshell; Arthropoda; Insecta; Lepidoptera; Nymphalidae). The genome sequence is 398 megabases in span. The majority of the assembly is scaffolded into 32 chromosomal pseudomolecules, with the W and Z sex chromosome assembled.

## Species taxonomy

Eukaryota; Metazoa; Ecdysozoa; Arthropoda; Hexapoda; Insecta; Pterygota; Neoptera; Endopterygota; Lepidoptera; Glossata; Ditrysia; Papilionoidea; Nymphalidae; Nymphalinae; Nymphalis; Nymphalis; *Nymphalis polychloros* (Linnaeus, 1758) (NCBI:txid171594).

## Introduction

The large tortoiseshell, also known as the black-legged tortoiseshell or elm nymphalid, is a widespread but rare butterfly in woodlands across continental Europe, North Africa and Central Asia. Once common in England and Wales, *N. polychloros* went extinct in Southern Britain in the 1960s for unknown reasons and is currently classified as ‘vulnerable’ in several European countries ( Maes *et al.*, 2020). It is listed as Least Concern in the IUCN Red List Category (Europe) ( van Swaay *et al.*, 2010). However, recent sightings of a breeding colony in Dorset in 2021 suggest that this species is once again resident in the UK. It is morphologically very close to both the small tortoiseshell, *Aglais urticae*, and the scarce tortoiseshell, *N. xanthomelas*, in adult appearance. The species uses a wide variety of host plants such as *Pyrus*, *Prunus*, *Salix*, *Ulmus*, *Crataegus*, and others. It is univoltine and overwinters as an adult. ( Lorković, 1941) reported a karyotype of 31 chromosomes and the genome size estimated for its relative, *Aglais io*, is 363.5 Mb ( Mackintosh *et al.*, 2019).

## Genome sequence report

**Figure 1.**
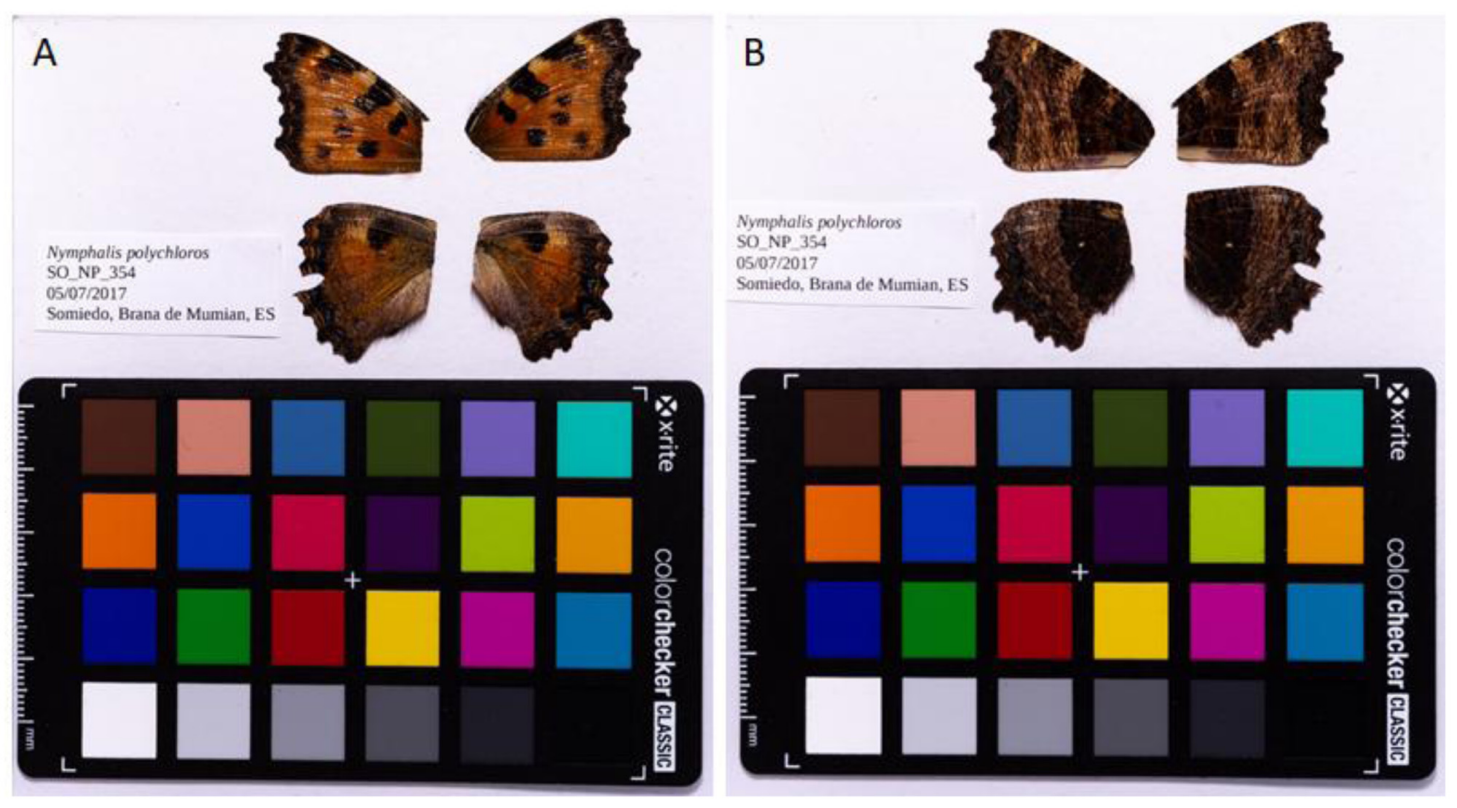
Fore and hind wings of *Nymphalis polychloros* specimen from which the genome was sequenced. ( **A**) Dorsal surface view of wings from specimen SO_NP_354 (ilNymPoly1) from Somiedo, Spain used to generate Pacific Biosciences and 10X genomics data. ( **B**) Ventral surface view of wings from specimen SO_NP_354 from Somiedo, Spain, used to generate Pacific Biosciences and 10X genomics data.

**Figure 2.**
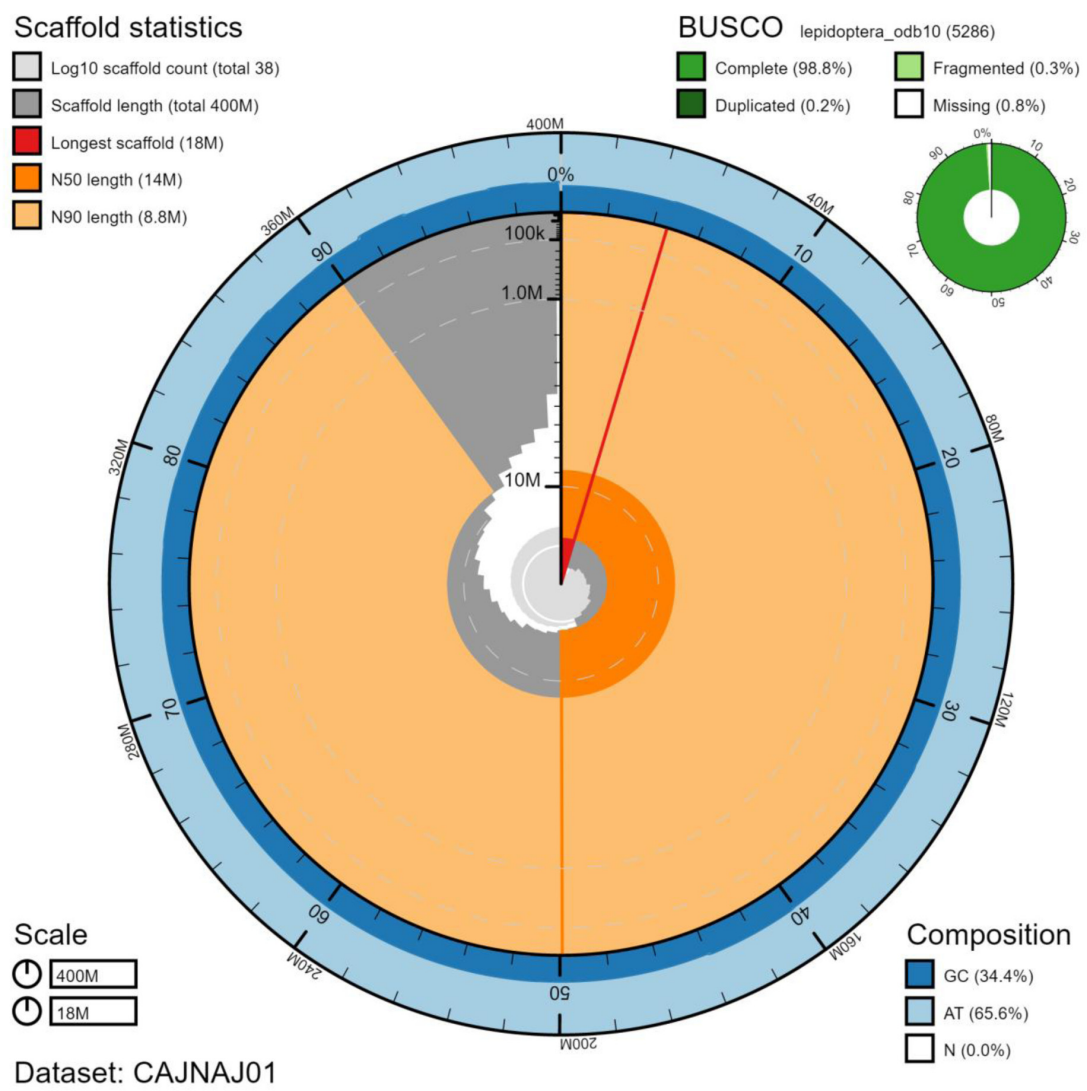
Genome assembly of *Nymphalis polychloros*, ilNymPoly1.1: metrics. The BlobToolKit Snailplot shows N50 metrics and BUSCO gene completeness. An interactive version of this figure is available at https://blobtoolkit.genomehubs.org/view/ilNymPoly1.1/dataset/CAJNAJ01/snail.

The genome was sequenced from a single female *N. polychloros* ( Figure 1) to 36-fold coverage in Pacific Biosciences single-molecule long reads and 84-fold coverage in 10X Genomics read clouds. Primary assembly contigs were scaffolded with chromosome conformation Hi-C data. Manual assembly curation corrected two missing/misjoins, reducing the scaffold number by 5.31%. The final assembly has a total length of 398 Mb in 38 sequence scaffolds with a scaffold N50 of 14 Mb ( Table 1). Of the assembly sequence, 100% was assigned to 32 chromosomal-level scaffolds, representing 30 autosomes (numbered by sequence length), and the W and Z sex chromosome ( Figure 2– Figure 5; Table 2). The assembly has a BUSCO v5.1.2 ( Simão *et al.*, 2015) completeness of 98.8% using the lepidoptera_odb10 reference set. While not fully phased, the assembly deposited is of one haplotype. Contigs corresponding to the second haplotype have also been deposited.

**Table 1.** Genome data for *Nymphalis polychloros*, ilNymPoly1.1.

*Project accession data*	
Assembly identifier	ilNymPoly1.1
Species	*Nymphalis polychloros*
Specimen	ilNymPoly1
NCBI taxonomy ID	NCBI:txid171594
BioProject	PRJEB43012
BioSample ID	SAMEA7523140
Isolate information	Female, whole organism
*Raw data accessions*	
PacificBiosciences SEQUEL II	ERR6590585
10X Genomics Illumina	ERR6054433-ERR6054436
Hi-C Illumina	ERR6054437
RNAseq PolyA Illumina	ERR6286714
*Genome assembly*	
Assembly accession	GCA_905220585.1
*Accession of alternate haplotype*	GCA_905220575.1
Span (Mb)	398
Number of contigs	45
Contig N50 length (Mb)	14
Number of scaffolds	38
Scaffold N50 length (Mb)	14
Longest scaffold (Mb)	17
BUSCO * genome score	C:98.8%[S:98.6%,D:0.2%],F:0.3%,M:0.8%,n:5286

*BUSCO scores based on the lepidoptera_odb10 BUSCO set using v5.1.2. C= complete [S= single copy, D=duplicated], F=fragmented, M=missing, n=number of orthologues in comparison. A full set of BUSCO scores is available at https://blobtoolkit.genomehubs.org/view/ilNymPoly1.1/dataset/CAJNAJ01/busco.

**Figure 3.**
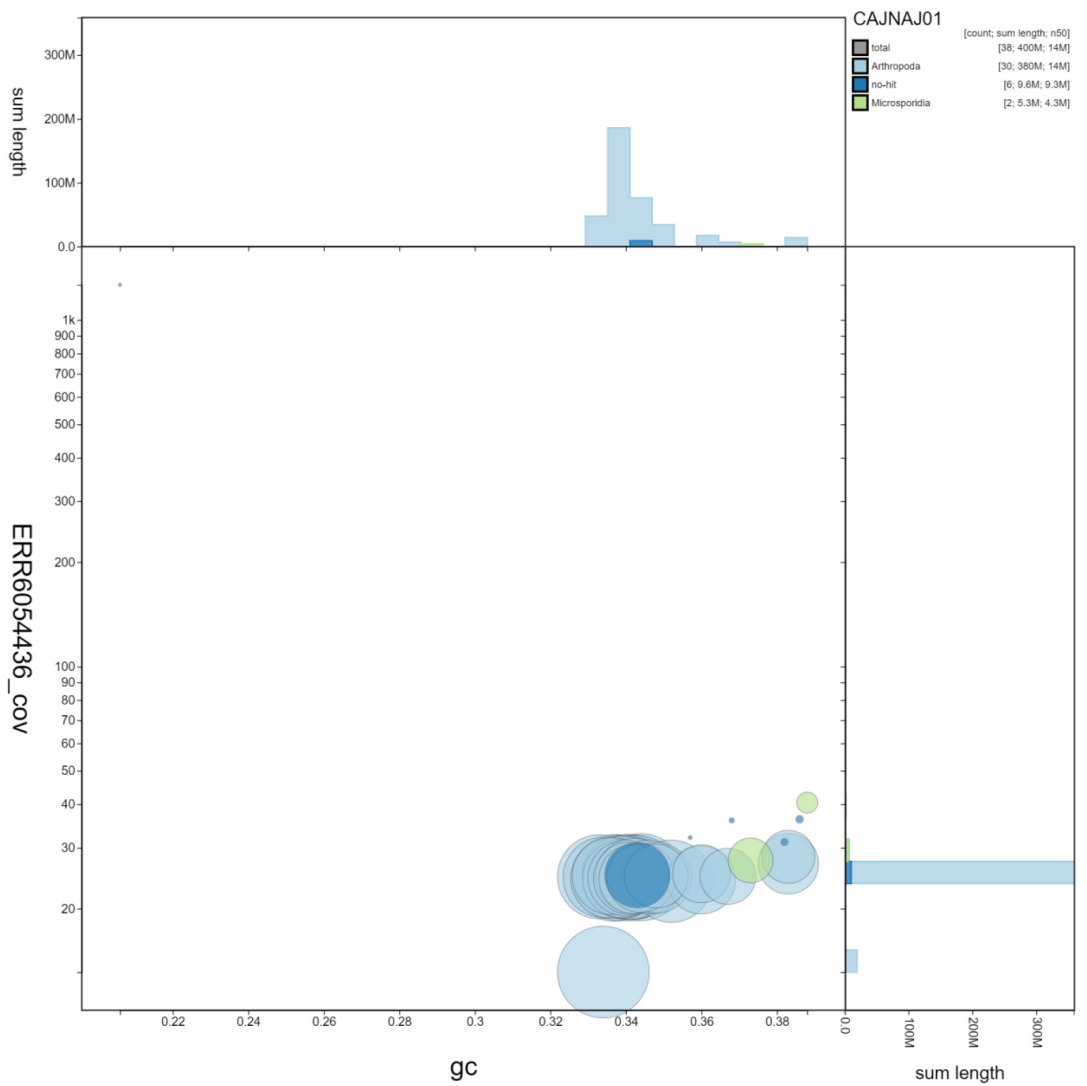
Genome assembly of *Nymphalis polychloros*, ilNymPoly1.1: GC coverage. BlobToolKit GC-coverage plot. Chromosomes are coloured by phylum. Circles are sized in proportion to chromosome length Histograms show the distribution of chromosome length sum along each axis. An interactive version of this figure is available at https://blobtoolkit.genomehubs.org/view/ilNymPoly1.1/dataset/CAJNAJ01/blob.

**Figure 4.**
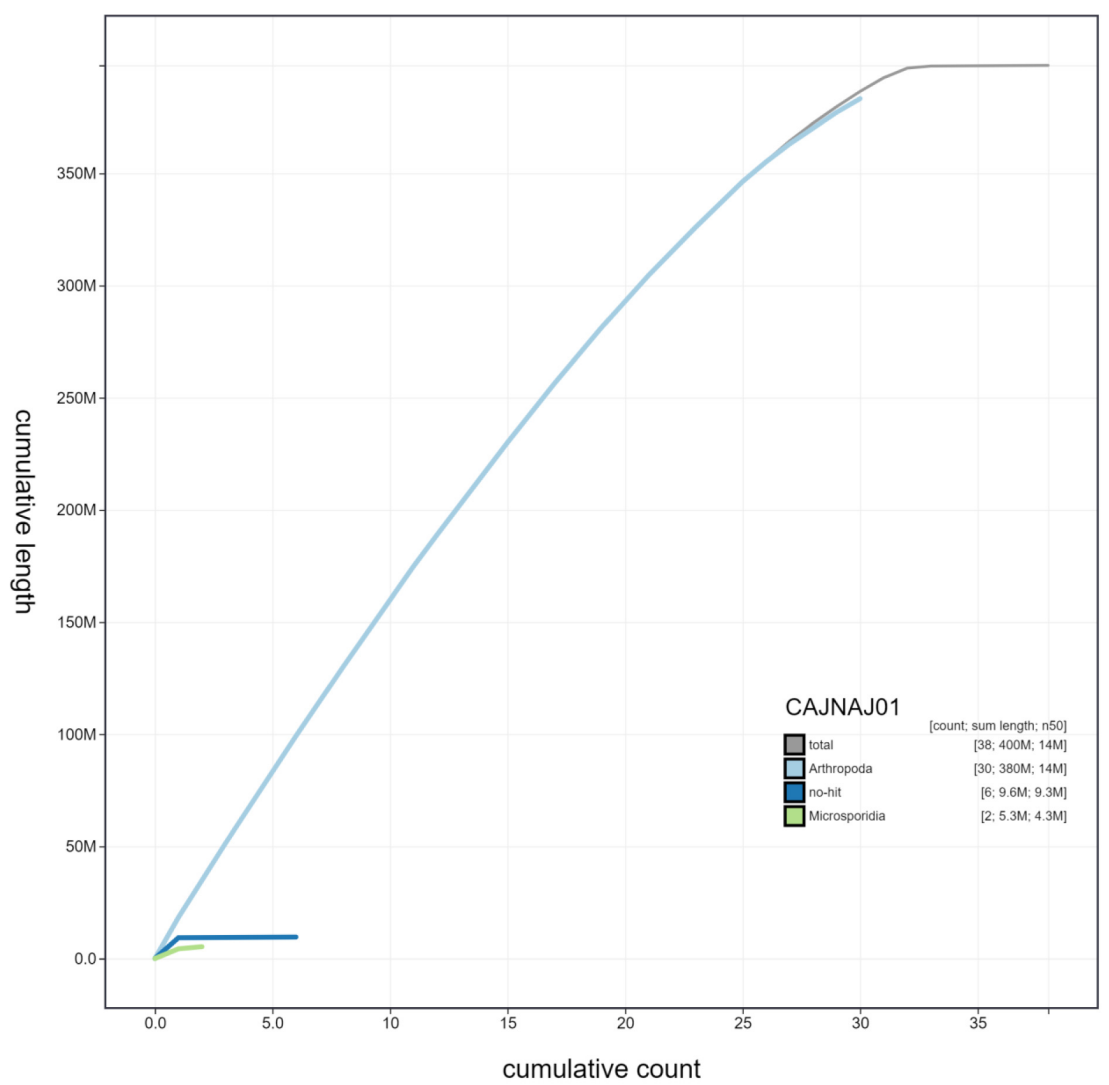
Genome assembly of *Nymphalis polychloros*, ilNymPoly1.1: cumulative sequence. BlobToolKit cumulative sequence plot. The grey line shows cumulative length for all chromosomes. Coloured lines show cumulative lengths of chromosomes assigned to each phylum using the buscogenes taxrule. An interactive version of this figure is available at https://blobtoolkit.genomehubs.org/view/ilNymPoly1.1/dataset/CAJNAJ01/cumulative.

**Figure 5.**
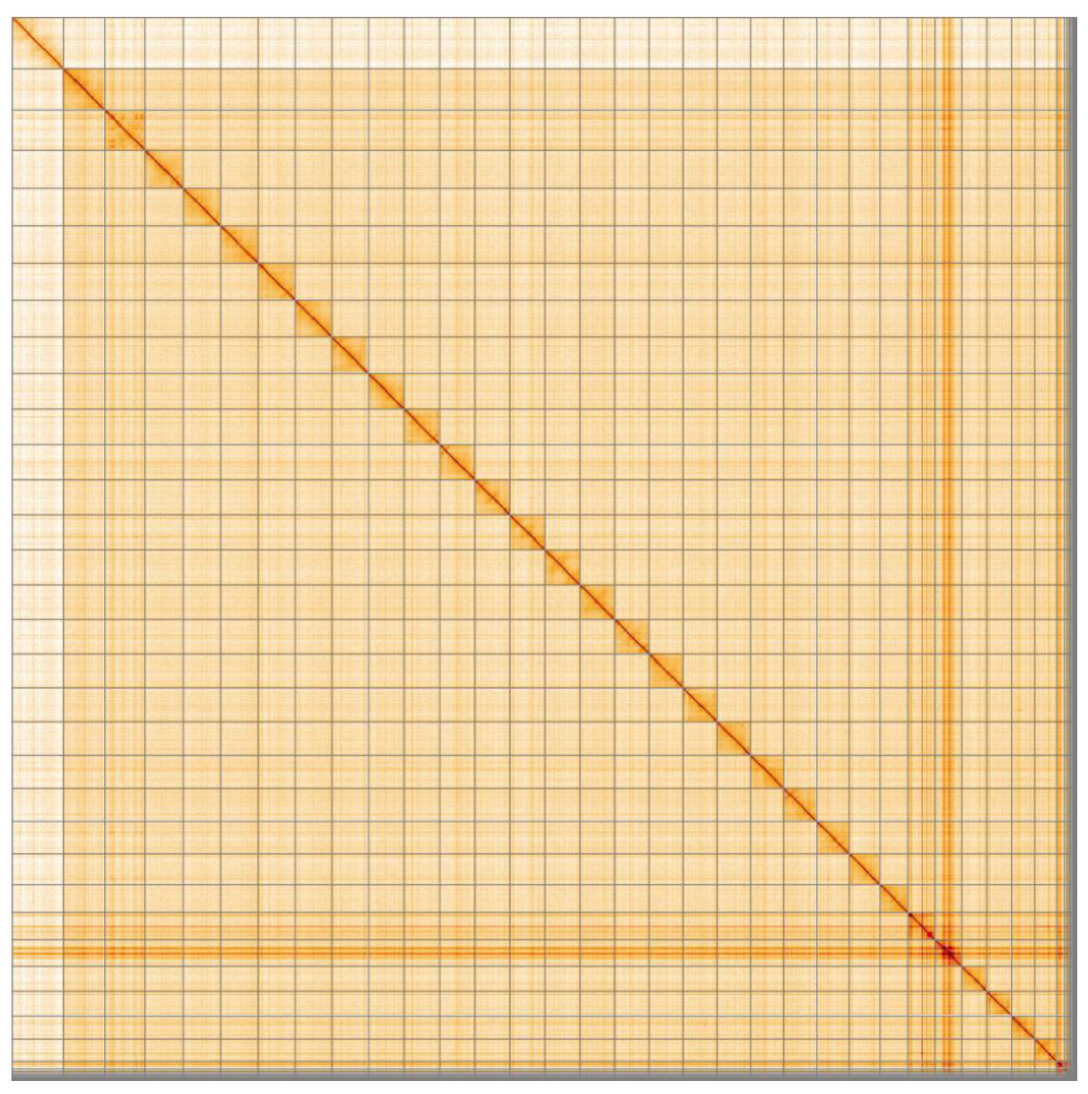
Genome assembly of *Nymphalis polychloros*, ilNymPoly1.1: Hi-C contact map. Hi-C contact map of the ilNymPoly1.1 assembly, visualised in HiGlass.

**Table 2.** Chromosomal pseudomolecules in the genome assembly of *Nymphalis polychloros*, ilNymPoly1.1.

INSDC accession	Chromosome	Size (Mb)	GC%
HG992242.1	1	16.56	34.4
HG992243.1	2	16.45	33.7
HG992244.1	3	16.03	34.1
HG992245.1	4	15.91	33.8
HG992246.1	5	15.83	34.1
HG992247.1	6	15.48	34
HG992248.1	7	15.41	33.3
HG992249.1	8	15.04	34.3
HG992250.1	9	14.99	34.1
HG992251.1	10	14.77	35.2
HG992252.1	11	14.06	33.4
HG992253.1	12	13.93	33.6
HG992254.1	13	13.74	33.9
HG992255.1	14	13.53	33.9
HG992256.1	15	13.45	33.9
HG992257.1	16	12.92	33.6
HG992258.1	17	12.55	34
HG992259.1	18	12.34	34.2
HG992260.1	19	11.88	34.6
HG992261.1	20	11.42	34.1
HG992262.1	21	10.92	34.8
HG992263.1	22	10.46	34.2
HG992264.1	23	10.26	34.4
HG992265.1	24	10.09	36
HG992266.1	25	9.27	34.3
HG992267.1	26	8.82	34.8
HG992268.1	27	7.95	38.3
HG992269.1	28	7.29	36
HG992270.1	29	6.82	36.7
HG992271.1	30	6.08	38.3
HG992272.1	W	4.33	37.3
HG992241.1	Z	18.34	33.4
HG992273.1	MT	0.02	20.3
-	Unplaced	1.22	38.6

## Methods

The female *N. polychloros* specimen SC_NP_345 was collected using a net from Somiedo, Brana de Mumian, Asturias, Spain (latitude 43.0679, longitude -6.239918) by Konrad Lohse, University of Edinburgh. Permissions for field sampling were granted by the Gobierno del Principado de Asturias (014252). The specimen was snap-frozen from live in liquid nitrogen.

DNA was extracted from thorax tissue at the Wellcome Sanger Institute (WSI) Scientific Operations core from the whole organism using the Qiagen MagAttract HMW DNA kit, according to the manufacturer’s instructions. RNA was extracted (also from thorax tissue) in the Tree of Life Laboratory at the WSI using TRIzol (Invitrogen), according to the manufacturer’s instructions. RNA was then eluted in 50 μl RNAse-free water and its concentration RNA assessed using a Nanodrop spectrophotometer and Qubit Fluorometer using the Qubit RNA Broad-Range (BR) Assay kit. Analysis of the integrity of the RNA was done using Agilent RNA 6000 Pico Kit and Eukaryotic Total RNA assay.

Pacific Biosciences HiFi circular consensus and 10X Genomics read cloud DNA sequencing libraries, in addition to PolyA RNA-Seq libraries, were constructed according to the manufacturers’ instructions. DNA and RNA sequencing was performed by the Scientific Operations core at the WSI on Pacific Biosciences SEQUEL II (HiFi), Illumina HiSeq X (10X) and Illumina HiSeq 4000 (RNA-Seq) instruments. Hi-C data were generated from abdomen tissue using the Arima v2.0 kit and sequenced on Illumina NovaSeq.

Assembly was carried out with Hifiasm ( Cheng *et al.*, 2021); haplotypic duplication was identified and removed with purge_dups ( Guan *et al.*, 2020). One round of polishing was performed by aligning 10X Genomics read data to the assembly with longranger align, calling variants with freebayes ( Garrison & Marth, 2012). The assembly was then scaffolded with Hi-C data ( Rao *et al.*, 2014) using SALSA2 ( Ghurye *et al.*, 2019). The assembly was checked for contamination and corrected using the gEVAL system ( Chow *et al.*, 2016) as described previously ( Howe *et al.*, 2021). Manual curation was performed using gEVAL, HiGlass ( Kerpedjiev *et al.*, 2018) and Pretext. The mitochondrial genome was assembled using MitoHiFi ( Uliano-Silva *et al.*, 2021). The genome was analysed and BUSCO scores generated within the BlobToolKit environment ( Challis *et al.*, 2020). Table 3 contains a list of all software tool versions used, where appropriate.

**Table 3.** Software tools used.

Software tool	Version	Source
Hifiasm	0.12	Cheng *et al.*, 2021
purge_dups	1.2.3	Guan *et al.*, 2020
longranger	2.2.2	https:// support.10xgenomics. com/genome-exome/ software/pipelines/latest/ advanced/other-pipelines
freebayes	1.3.1-17-gaa2ace8	Garrison & Marth, 2012
SALSA2	2.2	Ghurye *et al.*, 2019
MitoHiFi	1.0	Uliano-Silva *et al.*, 2021
gEVAL	N/A	Chow *et al.*, 2016
HiGlass	1.11.6	Kerpedjiev *et al.*, 2018
PretextView	0.1.x	https://github.com/wtsi-hpag/PretextView
BlobToolKit	2.6.1	Challis *et al.*, 2020

The materials that have contributed to this genome note were supplied by a Tree of Life collaborator. The Wellcome Sanger Institute employs a process whereby due diligence is carried out proportionate to the nature of the materials themselves, and the circumstances under which they have been/are to be collected and provided for use. The purpose of this is to address and mitigate any potential legal and/or ethical implications of receipt and use of the materials as part of the research project, and to ensure that in doing so we align with best practice wherever possible.

The overarching areas of consideration are:

- Ethical review of provenance and sourcing of the material;
- Legality of collection, transfer and use (national and international).

Each transfer of samples is undertaken according to a Research Collaboration Agreement or Material Transfer Agreement entered into by the Tree of Life collaborator, Genome Research Limited (operating as the Wellcome Sanger Institute) and in some circumstances other Tree of Life collaborators.

## Data availability

European Nucleotide Archive: Nymphalis polychloros (large tortoiseshell). Accession number PRJEB42956; https://identifiers.org/ena.embl:PRJEB42956.

The genome sequence is released openly for reuse. The *N. polychloros* genome sequencing initiative is part of the Darwin Tree of Life (DToL) project. All raw sequence data and the assembly have been deposited in INSDC databases.The genome will be annotated using the RNA-Seq data and presented through the Ensembl pipeline at the European Bioinformatics Institute. Raw data and assembly accession identifiers are reported in Table 1.
