# Supplementary material for: The genome sequence of the large tortoiseshell, Nymphalis polychloros (Linnaeus, 1758)
Source: Wellcome Open Res. 2021 Sep 16;6:238. [Version 1] doi: 10.12688/wellcomeopenres.17196.1 (PMC9039367; doi:10.12688/wellcomeopenres.17196.1)
Supplement: Peer review history

# Peer review: Reviewer response for version 1

**DOI:** 10.21956/wellcomeopenres.19000.r49891

**Published:** 2023-05-03

**Authors:** Arnaud Martin

**Affiliations:** 1 Department of Biological Sciences, The George Washington University, Washington, DC, USA

This report describes a near-complete genome assembly for the butterfly *Nymphalis polychloros, *which will be an excellent resource for the study of butterfly genetics and diversification. It is worth mentioning that a high-quality genome annotation is available for the closely related species Nymphalis (Aglais) io. I recommend mentioning Zhang et al. 2021, for its clarification of the genus status in the corresponding clade.

Are sufficient details of methods and materials provided to allow replication by others?

Yes

Is the rationale for creating the dataset(s) clearly described?

Yes

Are the datasets clearly presented in a useable and accessible format?

Yes

Are the protocols appropriate and is the work technically sound?

Yes

Reviewer Expertise:

Buttferly evolutionary genomics and development

I confirm that I have read this submission and believe that I have an appropriate level of expertise to confirm that it is of an acceptable scientific standard.

## References

[1] : Genomics-guided refinement of butterfly taxonomy. *Taxon Rep Int Lepid Surv* .2021;9: 10.5281/zenodo.5630311 10.5281/zenodo.5630311 35098146PMC8794009

# Peer review: Reviewer response for version 1

**DOI:** 10.21956/wellcomeopenres.19000.r49892

**Published:** 2022-04-25

**Authors:** Jiasheng Hao

**Affiliations:** 1 Laboratory of Molecular Evolution and Biodiversity, Anhui Normal University, Wuhu, China

1. In the last sentence of the manuscript's Introduction, "(Lorković, 1941) reported a karyotype of 31 chromosomes and the genome size estimated for its relative, *Aglais io*, is 363.5Mb (Mackintosh *et al.*, 2019).”We see that the genome size of *Nymphalis polychloros* has not been reported in previous studies. It is suggested that the authors should increase the analysis of genome size prediction based on GCE or other tools in the Methods, and this is a good guide for subsequent genome assembly or haplotypic selection.
2. In the part of “Genome sequence report”, the W and Z sex chromosomes were mentioned to be assembled in this work. However, how to identify sex chromosomes in the Methods was not stated clearly, and the authors are suggested to clearly introduce the method for distinguishing sex chromosomes in this study.
3. The results of genome assembly probably depend on the setting of parameters used in the software, thus the authors are suggested to add the main parameters of tools in Table 3.
4. For some quality control analysis in the Methods, can the authors describe the changes in data volume before and after filtering in the text? For example, “haplotypic duplication was identified and removed with purge_dups (Guan *et al.*, 2020).”  “Manual curation was performed using Manual curation was performed using gEVAL, HiGlass (Kerpedjiev *et al.*, 2018) and Pretext.”

Are sufficient details of methods and materials provided to allow replication by others?

Yes

Is the rationale for creating the dataset(s) clearly described?

Yes

Are the datasets clearly presented in a useable and accessible format?

Yes

Are the protocols appropriate and is the work technically sound?

Yes

Reviewer Expertise:

the phylogeny and evolutionary history of some butterfly groups.

I confirm that I have read this submission and believe that I have an appropriate level of expertise to confirm that it is of an acceptable scientific standard.

# Peer review: Reviewer response for version 1

**DOI:** 10.21956/wellcomeopenres.19000.r45905

**Published:** 2021-10-18

**Authors:** Mathieu Joron, Quentin Rougemont

**Affiliations:** 1 Centre d'Ecologie Fonctionnelle et Evolutive (CEFE), CNRS, Université de Montpellier, Montpellier, France

This data note by Lohse and colleagues reports the genome sequence of a butterfly in the large family Nymphalidae, well known for harbouring other iconic butterfly models for metapopulation dynamics or for the study of wing coloration. This adds a reference genome for a group of butterflies with interesting biology such as adult overwintering and long distance migration.

 The genome presented is of very high quality. I see nothing problematic with the assembly which offers excellent contiguity. My comments are minor but may reflect the perspective of someone involved in assembling similar genomes.

 It may make no difference, but wondered why the length was initially estimated to the 363Mb of the peacock butterfly *Aglais io*, while the comma butterfly *Polygonia c-album* appears more closely related, and has a published, slightly higher genome size estimated to 373Mb (Celorio-Mancera *et al. *2021 Genome Biol. Evol. 13:evab054) ^1^

 Table 2 reports 1.22Mb of unplaced scaffolds, and the text reports 38 sequence scaffolds. Yet the authors report that 100% of the assembly was assigned to 32 chromosomal-level scaffolds. I was wondering what constitutes the difference between those statistics (large heterozygous tracts/haplotypes?)

 Hi-C data was generated from abdomen tissue. Since this is a wild and therefore mated female, the abdomen is likely to contain recombinant gametes from one or several unknown males. I assume that this is unlikely to introduce large errors for scaffolding, but wondered whether the authors could perhaps give a few words on this possible issue, since it is a question that frequently arises when using abdominal female tissue from wild-caught individuals.

 This assembly is made from the DNA of a wild-caught specimen. Therefore it would be interesting to provide details on its observed heterozygosity. Dealing with heterozygosity is a recurrent issue in genome assembly.

 Similarly, chosen parameter values would be good to provide for all packages and softwares (such as hifiasm, freebase, etc), perhaps as an additional column for table 3. Those values are essential for reproducibility, but also very useful for people assembling similar genomes.

 It would be interesting to provide details of the improvements allowed by the different steps (for instance the polishing step, by giving stats before and after). Again this would be useful for other users and generally for assembly of similar genomes.  This could take the form of a table.

 The note presents the generation of RNAseq data, which is great, but the data is not (yet) used for annotation. I wondered why include this in the methods if it is actually not analysed.

 Status/justification: This species the large tortoiseshell is a fairly common though elusive butterfly in its predominantly continental European range.  I understand that the Darwin tree of life effort is motivated by sequencing “British taxa” and the status of this species in Britain may have influenced its position on the priority list. However, the aim of a reference genome probably goes beyond that. Abundance and conservation status of taxa are very variable depending on how far from the range margin one stands! From a broader perspective, the large tortoiseshell is a forest species with a broad European distribution. It has a relatively poorly known ecology compared to closely related species. And interesting question marks remain regarding the origins and status of its genetic structure (vicariance, speciation?). *Nymphalis *as a genus also has unclear relationships with other genera such as *Polygonia *and *Kaniska*. Perhaps a reference genome could stimulate interesting research on those aspects which could make a better "justification" for sequencing it than the recent sighting of a colony in Dorset where the species is teetering on its range margin.

Are sufficient details of methods and materials provided to allow replication by others?

Partly

Is the rationale for creating the dataset(s) clearly described?

Yes

Are the datasets clearly presented in a useable and accessible format?

Yes

Are the protocols appropriate and is the work technically sound?

Yes

Reviewer Expertise:

Population genomics

We confirm that we have read this submission and believe that we have an appropriate level of expertise to confirm that it is of an acceptable scientific standard.

## References

[1] : Chromosome Level Assembly of the Comma Butterfly (Polygonia c-album ). *Genome Biology and Evolution* .2021;13(5) : 10.1093/gbe/evab054 10.1093/gbe/evab054 PMC814020533749729
